# New Remedies

**Published:** 1908-09-12

**Authors:** 


### New Remedies.

In the matter of the treatment of disease by drugs it is possible to distinguish at the present time three main groups of physicians. Among other characteristics each group adopts a different attitude towards the new remedies which are daily being brought to the notice of the medical profession. Firstly, there are those physicians who, while refusing to consider drugs as the only means of treatment, yet believe that a thorough knowledge of the resources of the materia medica is essential to sound and successful therapeutics. In their opinion the ideal method of medical treatment consists in an orderly and flexible application of regimen, nursing, and medication. In this school may be ranked those physicians who rely for the most part upon a few standard drugs, whose value and resources have been proved in their own practice and in the experience of their teachers. Generally speaking, they are sceptical of new remedies with complex chemical formulæ and alluring titles, and are often heard to say that the old and tried drugs are sufficient for all needs in the hands of those who know how to use them. They assert that, with one or two exceptions, there have been no recent additions to the pharmacopœia of any permanent value.

With this school we must confess that we are largely in sympathy. We cannot believe that drugs will ever be relegated to an insignificant position in the stock-in-trade of the physician. We altogether decline to associate ourselves with the more advanced members of the second group of practitioners —the so-called "therapeutic nihilists"—who seem to anticipate a time when the pharmacopœia and all its contents will be banished to the shelves of the museum, and who indeed (if we are correctly informed) are sceptical of any therapeutic measures which aim at replacing, or even supplementing, the *vis medicatrix naturæ*. We believe that no advances in medical science will ever do more than reorganise the pharmacopœia. Many drugs are no doubt useless; many, indeed, may be prejudicial to the conditions for which they are prescribed; but there is an irreducible minimum, without which the physician will ever be imperfectly equipped for the treatment of his patients. The fact that hundreds of drugs are misused, and that many hundreds more might well be abolished from the pharmacopœia, will not undermine our confidence in those standard remedies which have stood the test of time, or lead us to despair of the discovery of new and valuable additions to our catalogue of medicines.

The third group of physicians stands in direct contrast to the "therapeutic nihilists." Here are to be found those practitioners upon whom all drugs —and especially those which are new and of synthetic origin and of Teutonic manufacture—seem to exercise a peculiar fascination. Physicians of this temperament abound upon the Continent, and they are not unknown in America; while in this country their existence can at least be conjectured. In their attitude towards new remedies we may discern two opposite qualities: one which compels our admiration, and one which we can only deplore. While we respect the enterprising spirit which bids them try all things in their confident search for therapeutic improvement, we must condemn the restless credulity with which they embrace yesterday's unfledged products of the foreign laboratory, to the neglect of those old and well-tried remedies whose manifold uses are only revealed to the persevering and the resourceful. We would never advocate "contempt prior to examination" in matters therapeutical: medical treatment in all its branches can only be the better for enterprise and scientific experimentation. But there is room for caution and common sense and scepticism in our attitude towards all new remedies, and particularly towards those whose claims, although cloaked in the language of science and addressed to the medical profession alone, are scarcely less preposterous than those of the products of quackery.

